# Genetic variations in histidine-rich protein 2 and histidine-rich protein 3 of Myanmar *Plasmodium falciparum* isolates

**DOI:** 10.1186/s12936-020-03456-6

**Published:** 2020-11-02

**Authors:** Hương Giang Lê, Jung-Mi Kang, Jinyoung Lee, Won Gi Yoo, Moe Kyaw Myint, Khin Lin, Tong-Soo Kim, Byoung-Kuk Na

**Affiliations:** 1 Department of Parasitology and Tropical Medicine and Institute of Health Sciences, Gyeongsang National University College of Medicine, Jinju, 52727 Republic of Korea; 2 Department of Convergence Medical Science, Gyeongsang National University, Jinju, 52727 Republic of Korea; 3 Department of Tropical Medicine and Inha Research Institute for Medical Sciences, Inha University College of Medicine, Incheon, 22212 Republic of Korea; 4 Korea Mouse Phenotyping Center, Seoul National University, Seoul, 08826 Republic of Korea; 5 Department of Medical Research Pyin Oo Lwin Branch, Pyin Oo Lwin, Myanmar

**Keywords:** *Plasmodium falciparum*, Histidine-rich protein 2, Histidine-rich protein 3, Myanmar, Genetic polymorphism

## Abstract

**Background:**

Malaria rapid diagnostic tests (RDTs) are precious tools to diagnose malaria. Most RDTs used currently are based on the detection of *Plasmodium falciparum* histidine-rich protein 2 (PfHRP2) in a patient’s blood. However, concern has been raised in recent years that deletion of *pfhrp2* in the parasite could affect the accuracy of PfHRP2-based RDTs. In addition, genetic variation in *pfhrp2* might influence the accuracy and sensitivity of RDTs. In this study, the genetic variation in *pfhrp2* and *pfhrp3* in Myanmar *P. falciparum* isolates was analysed.

**Methods:**

Blood samples were collected from malaria patients who were infected with *P. falciparum* in Mandalay, Naung Cho, Tha Beik Kyin, and Pyin Oo Lwin, Upper Myanmar between 2013 and 2015. The *pfhrp2* and *pfhrp3* were amplified by nested polymerase chain reaction (PCR), cloned and sequenced. Genetic variation in Myanmar *pfhrp2* and *pfhrp3* was analysed using the DNASTAR program. Comparative analysis of Myanmar and global *pfhrp2* and *pfhrp3* isolates was also performed.

**Results:**

One-hundred and two *pfhrp2* and 89 *pfhrp3* were amplified from 105 blood samples, of which 84 *pfhrp2* and 56 *pfhrp3* sequences were obtained successfully. Myanmar *pfhrp2* and *pfhrp3* showed high levels of genetic variation with different arrangements of distinct repeat types, which further classified Myanmar *pfhrp2* and *pfhrp3* into 76 and 47 haplotypes, respectively. Novel amino acid changes were also found in Myanmar *pfhrp2* and *pfhrp3*, but their frequencies were very low. Similar structural organization was shared by Myanmar and global *pfhrp2* and *pfhrp3*, and differences in frequencies of repeat types and lengths were also observed between and among global isolates.

**Conclusion:**

Length polymorphisms and amino acid substitutions generated extensive genetic variation in Myanmar *pfhrp2* and *pfhrp3*. Comparative analysis revealed that global *pfhrp2* and *pfhrp3* share similar structural features, as well as extensive length polymorphisms and distinct organizations of repeat types. These results provide a better understanding of the genetic structure of *pfhrp2* and *pfhrp3* in global *P. falciparum* populations and suggest useful information to develop RDTs with improved quality.

## Background

Myanmar has the majority of malaria cases and deaths in Southeast Asia, but the incidence of the disease has declined dramatically during the last decade and is progressing steadily towards elimination [1]. The annual number of malaria cases in the country has dropped from approximately 700,000 in 2010 to about 85,000 in 2017 [1]. Microscopic examination of a blood smear is the primary diagnostic tool for malaria in Myanmar, but malaria rapid diagnostic tests (RDTs) have become a valuable alternative for use in remote areas where microscopy may not be feasible or where microscopy results would not be available immediately. RDTs offer a practical alternative to microscopy because they do not require a laboratory or special equipment, are simple to use, and provide reliable results in a short time [2, 3]. RDTs have been introduced as reliable diagnostic tools in many malaria-endemic areas, including Myanmar.

Malaria RDTs are designed to detect one or more *Plasmodium* antigens in a patient’s blood by using specific monoclonal antibodies. Several antigens of *Plasmodium falciparum* have been utilized in RDTs for malaria detection, including histidine-rich protein 2 (PfHRP2), lactate dehydrogenase (PfLDH) and aldolase [4]. Among these antigens, PfHRP2 is the most widely employed in commercially available malaria RDTs at present, because of its abundant expression in the asexual blood stage of *P. falciparum* [5–7], structural stability [8] and high specificity recognized by multiple antibodies [9–11]. PfHRP2 is a protein encoded by *pfhrp2*, which is located in the sub-telomeric region of chromosome 8. This *P. falciparum*-specific protein is expressed abundantly in the infected erythrocyte surface of the blood stage and early gametocyte stage [12, 13]. Some of the PfHRP2-based RDTs can cross-react with a structural homologue, *P. falciparum* histidine-rich protein 3 (PfHRP3) that is encoded by *pfhrp3* and shares high sequence identity and epitope similarity with PfHRP2 [13, 14]. The *pfhrp2* and *pfhrp3* genes are predicted to be derived from a common ancestral gene. As such, these near-duplicate genes may compensate each other in function [7]. However, it has been reported recently that *P. falciparum* field isolates in some parts of malaria-endemic regions lack *pfhrp2* [15–20]. Deletion of *pfhrp2* could affect the accuracy of PfHRP2-based RDTs and lead to false-negative results followed by inappropriate treatment, which in turn causes negative impact for effective control and elimination of malaria. Furthermore, co-deletion of *pfhrp2* and *pfhrp3* has also been identified [8, 21]. In addition to the density of the parasite or the lack of PfHRP2 expression, it has also been suggested that genetic diversity in *pfhrp2* and *pfhrp3* could affect the sensitivity of PfHRP-based RDTs [22, 23]. Therefore, monitoring parasite factors that can affect performance of RDT-based diagnosis is important.

In this study, the genetic variation in *pfhrp2* and *pfhrp3* of Myanmar *P. falciparum* isolates was analysed. The diversity of the two genes from global *P. falciparum* isolates was also investigated comparatively to gain in-depth understanding of the genetic diversity and population structure of global *pfhrp2* and *pfhrp3*.

## Methods

### Study sites and blood sample collection

A total 105 blood samples from malaria patients infected with *P. falciparum* were collected during a previous study conducted in Myanmar between 2013 and 2015 [24]. The patients were detected during regional malaria surveys, which were conducted in the regions of Naung Cho, Pyin Oo Lwin, Tha Beik Kyin, and Mandalay in Upper Myanmar (Additional file 1: Fig. S1). Malaria infection was diagnosed by microscopic analysis of thin and thick blood smears. Finger-prick blood was taken from *P. falciparum*-infected patients and spotted in Whatman 3MM filter (GE Healthcare, Maidstone, UK) for confirmation by polymerase chain reaction (PCR) targeting 18S ribosomal RNA (rRNA) gene [24]. Informed consent was obtained from all patients before blood collection. The study protocol was approved by either the Ethics Committee of the Ministry of Health, Myanmar (97/Ethics 2015) or the Biomedical Research Ethics Review Board of Inha University School of Medicine, Republic of Korea (INHA 15-013).

### Genomic DNA extraction and amplifications of *pfhrp2* and *pfhrp3*

Genomic DNA was extracted from the dried blood spots using the QIAamp DNA Blood Kit (Qiagen, Hilden, Germany) following the manufacturer’s instructions. The primers specific for *pfhrp2* and *pfhrp3* were designed and used (Additional file 2: Table S1). Both genes were amplified by nested PCR methods. Each PCR was done with thermal cycling conditions: 94 °C for 5 min, and 35 cycles of 94 °C for 1 min, 53 °C for 1 min, and 72 °C for 1.5 min, followed by the final extension at 72 °C for 10 min. In order to minimize the nucleotide mis-incorporation during amplification, Ex *Taq* DNA polymerase (Takara, Otsu, Japan), which has a proof-reading activity, was used in all PCR steps. Each PCR product was resolved on 1.5% agarose gel, extracted from the gel, and cloned into T&A cloning vector (Real Biotech Corporation, Banqiao, Taiwan). Each ligation mixture was transformed into *Escherichia coli* DH5α competent cells. Colony PCR was performed to select the positive clones with appropriate inserts. The nucleotide sequences of the cloned *pfhrp2* and *pfhrp3* were analysed by automatic DNA sequencing with M13 forward and M13 reverse primers by the Sanger method. Plasmids from at least two independent clones from each transformation mixture were sequenced to verify the sequence accuracy. The nucleotide sequences of Myanmar *pfhrp2* and *pfhrp3* analysed in this study have been deposited in the GenBank database under the accession numbers KX138275–KX138311, MG417056–MG417080, and MT591418–MT591439 for *pfhrp2* and KX138312–KX138340 and MT591440–MT591466 for *pfhrp3*.

### Sequence analyses of Myanmar and global *pfhrp2* and *pfhrp3*

The nucleotide and deduced amino acid sequences of Myanmar *pfhrp2* and *pfhrp3* were analysed using EditSeq and SeqMan in the DNASTAR package (DNASTAR, Madison, WI, USA). Genetic variations of *pfhrp2* and *pfhrp3* in global *P. falciparum* isolates were also analysed. The *pfhrp2* and *pfhrp3* sequences deposited in public database were used in this study. The *pfhrp2* sequences analysed in this study were from China, India, Sri Lanka, Thailand, Philippines, Cambodia, Vietnam, Central African Republic, Ghana, Haiti, Kenya, Madagascar, Nigeria, Tanzania, French Guinea, Brazil, Honduras, Papua New Guinea, Solomon Islands, and East Timor (Additional file 3: Table S2). For *pfhrp3*, the sequences from Cambodia, India, Philippines, Kenya, Madagascar, Nigeria, Peru, Colombia, Papua New Guinea, and Solomon Islands were analysed (Additional file 4: Table S3).

## Results

### Amplification of *pfhrp2* and *pfhrp3* in Myanmar *Plasmodium falciparum* isolates

PCR amplification of *pfhrp2* and *pfhrp3* from 105 *P. falciparum*-infected blood samples resulted in successful amplification of 102 *pfhrp2* and 89 *pfhrp3*. The approximate sizes of amplified products were highly variable, ranging 100–1000 bp for *pfhrp2* and 50–600 bp for *pfhrp3*. Of these, 84 *pfhrp2* and 56 *pfhrp3* PCR products were cloned successfully and sequenced for further assessments. The remaining 19 *pfhrp2* and 33 *pfhrp3* PCR products were excluded from this study because the quality of the amplicons was not adequate for further analysis, despite repeated attempts.

### Polymorphic character of Myanmar *pfhrp2*

**Fig. 1:**
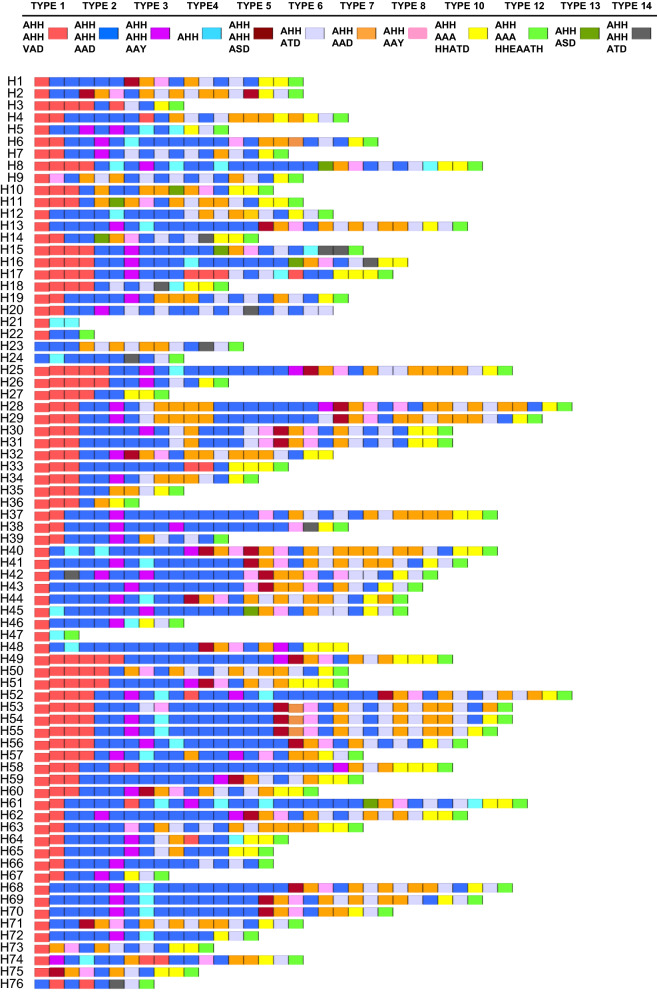
Genetic variation in Myanmar *pfhrp2.* Seventy-six unique haplotypes were identified in Myanmar *pfhrp2*. The haplotypes differ in the number and organization of 12 distinct repeat types. Each repeat type is displayed as a different color

Seventy-six distinct haplotypes of *pfhrp2* were identified in 84 Myanmar *P. falciparum* isolates (Fig. 1). Each haplotype consisted of different numbers of repeats ranging from 3 to 36. Different arrangements of distinct repeat types resulted in size variation between and among haplotypes. Haplotype 13 (H13) showed the highest prevalence with 4.76% (4/84), followed by haplotype 55 (H55), accounting for 3.57% (3/84). Most Myanmar *pfhrp2* started with 1–6 copies of type 1 repeat (AHHAHHVAD, 96.4%) and terminated with type 12 repeat (AHHAAAHHEAATH, 94%). However, three *pfhrp2* haplotypes (H23, H24, H76) began with type 2 repeat (AHHAHHAAD) and finished with type 12 repeat. Five haplotypes (H16, H20, H21, H32, H48) also started with type 1 repeat, but they terminated with type 4 (AHH), type 6 (AHHATD), or type 10 (AHHAAAHHATD).

### Polymorphic character of Myanmar *pfhrp3*

**Fig. 2:**
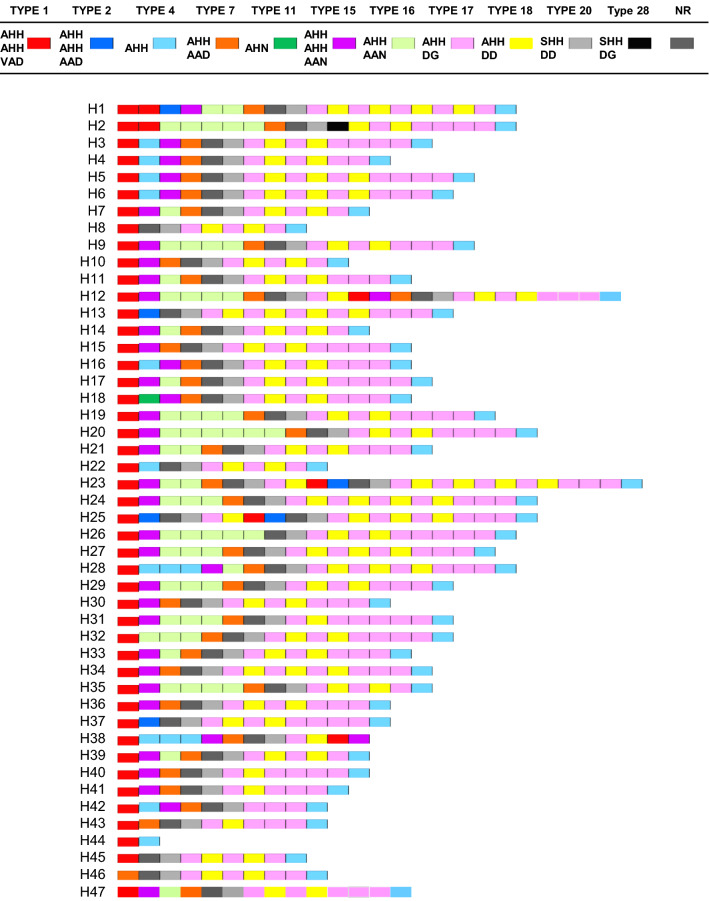
Genetic variation in Myanmar *pfhrp3.* Forty-seven unique haplotypes were identified in Myanmar *pfhrp3*. The haplotypes differ in the number and organization of 11 distinct repeat types. Each repeat type is displayed as a different color

Forty-seven distinct haplotypes of *pfhrp3* were observed in 56 Myanmar *P. falciparum* isolates (Fig. 2). Eleven different types of repeat were observed in Myanmar *pfhrp3* and each haplotype was constructed with different numbers of the repeats ranging from 2 to 25. Structural features of Myanmar *pfhrp3* haplotypes were highly diverse, but all haplotypes shared similar patterns. Most haplotypes started with type 1 repeat (AHHAHHVAD) and terminated with type 4 repeat (AHH). Two exceptions were haplotypes 38 and 46. Haplotype 38 stared with type 1 repeat but finished with type 15 repeat (AHHAHHAAN). Haplotype 46 began with type 7 repeat (AHHAAD) and terminated with type 4 repeat (AHH). Non-repeat (NR) regions were scattered randomly in the sequences of most haplotypes. The length variation in Myanmar *pfhrp3* was caused mainly by repeating numbers of type 16 (AHHAAN), type 17 (AHHADG), or type 18 (AHHDD).

### Amino acid changes in Myanmar *pfhrp2* and *pfhrp3*

Amino acid substitutions were identified in repeat types 1, 2, 6, and 7 of Myanmar *pfhrp2* (Table 1). Six variants of type 1 (AHHAHHVA**Y**, AH**R**AHHVAD, AHHA**R**HVAD, AH**P**AHHVAD, AHHAHH**E**AD, A**R**HAHHVAD) and six variants of type 2 (**V**HHAHHAAD, AHHAHHAA**G**, AHHAHH**T**AD, AHHA**R**HAAD, AHH**T**HHAAD, A**R**HAHHAAD) were found in Myanmar *pfhrp2*. Four variants of type 6 (**V**HHATD, AHHA**I**D, **D**HHATD, AHHA**P**D) were found. Two variants of type 7 were identified, in which one variant had a deletion of alanine (─HHAAD). The frequency of each variant in Myanmar *pfhrp2 *was low, ranging from 1.17 to 2.35%. For Myanmar *pfhrp3*, an amino acid change was also observed in each type 16 (AHHA**S**N), type 18 (A**R**HAAD), and type 20 (S**Y**HDD), but their frequencies in Myanmar *pfhrp3 *were also low (Table 1).

**Table 1:** The amino acid changes identified in repeat types in Myanmar *pfhrp2* and *pfhrp3*

Gene	Types	Variants	Frequency (%)
*pfhrp2*	Type 1 AHHAHHVAD	AHHAHHVA**Y**	1.17
		AH**R**AHHVAD*	1.17
		AHHA**R**HVAD*	1.17
		AH**P**AAHVAD*	1.17
		AHHAHH**E**AD*	1.17
		A**R**HAHHVAD*	1.17
	Type 2 AHHAHHAAD	**V**HHAHHAAD*	1.17
		AHHAHHAA**G**	2.35
		AHHAHH**T**AD	1.17
		AHHA**R**HAAD*	1.17
		AHH**T**HHAAD	2.35
		A**R**HAHHAAD	1.17
	Type 6 AHHATD	**V**HHATD*	1.17
		AHHA**I**D*	1.17
		**D**HHATD*	1.17
		AHHA**P**D	1.17
	Type 7 AHHAAD	HHAAD*	1.17
		AHHAAA*	1.17
*pfhrp3*	Type 16 AHHAAN	AHHA**S**N	1.78
	Type 18 AHHAAD	A**R**HAAD*	1.78
	Type 20 SHHDD	S**Y**HDD*	1.78

The amino acid changes are presented as bold. Asterisks indicate novel variants that have not been reported previously. The **–** in type 6 of *pfhrp2* means a deletion of alanine (A) at the corresponding position. Frequency means percentage of sequences with the corresponding repeat type variant in Myanmar *pfhrp2* or *pfhrp3*

### Prevalence of repeat types in Myanmar *pfhrp2* and *pfhrp3*

**Fig. 3:**
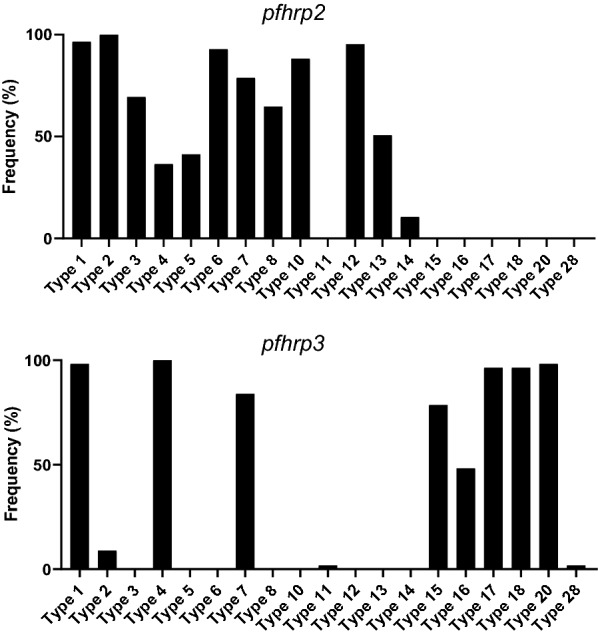
Frequencies of repeat types found in Myanmar *pfhrp2* and *pfhrp3.* The frequency of each repeat type identified in Myanmar *pfhrp2* and *pfhrp3* is presented

Overall prevalence of each type of repeat differed in Myanmar *pfhrp2* and *pfhrp3*. Type 2 repeat was the most prevalent and showed high numbers of duplicates in most haplotypes of Myanmar *pfhrp2*. Types 1, 6 and 12 were found in most haplotypes of Myanmar *pfhrp2*, accounting for 96.5, 92.9 and 95.3% of sequences, respectively. Type 4, 5, and 14 repeats were observed in only a few haplotypes of Myanmar *pfhrp2* and with lower prevalence (Fig. 3). For Myanmar *pfhrp3*, types 1, 4, 17, 18, and 20 repeats were found commonly in most haplotypes, while the proportions of other types of repeats were variable (Fig. 3). Types 1, 2, 4, and 7 repeats were found in both Myanmar *pfhrp2* and *pfhrp3*. Types 3, 5, 6, 8, 10, 12, 13, and 14 were identified only in Myanmar *pfhrp2* and types 11, 15, 16, 17, 18, 20, and 28 were found only in Myanmar *pfhrp3*.

### Genetic polymorphisms of *pfhrp2* and *pfhrp3* in global isolates

**Fig. 4:**
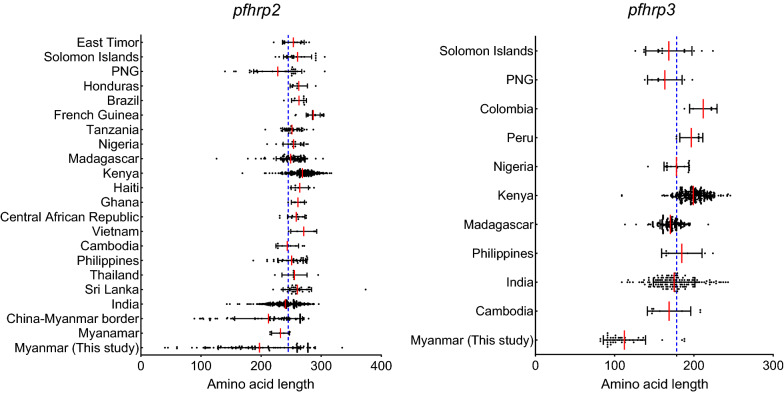
Comparison of length polymorphisms in global *pfhrp2* and *pfhrp3* isolates. The blue dotted line indicates the mean amino acid length in global *pfhrp2* and *pfhrp3*. The red lines represent the mean amino acid lengths of *pfhrp2* and *pfhrp3* calculated in *P. falciparum* isolates from each country

Comparative analysis of repeat types revealed that types 1, 2, 3, 5, 6, 7, 10, and 12 were found commonly in all global *pfhrp2* population (Table 2). The frequencies of types 1, 2, 6, and 12 were especially high in global *pfhrp2*. The frequencies of types 3, 5, 7, and 10 differed by country. Type 8 repeat also showed high frequencies in global *pfhrp2*, but it was not identified in Brazil *pfhrp2*. Types 4, 13 and 14 were observed at low frequencies in *pfhrp2* from some countries, but types 15 and type 19 were found only in *pfhrp2* from China, India, Kenya, or the Solomon Islands. A similar variety of frequencies of repeat types was found in global *pfhrp3* populations (Table 3). Types 1, 4, 7, 17, 18, and 20 were detected at high frequencies in all global *pfhrp3*. Type 16 also showed high frequencies in all global *pfhrp3*, except for Myanmar. Type 15 was found in nearly all global *pfhrp3* at relatively high frequencies, but it was not detected in *pfhrp3* from Peru, Papua New Guinea and the Solomon Islands. Types 2, 28 and 29 were identified at low frequencies in only several countries, including Myanmar, India, Philippines, Kenya, Madagascar, Peru, or Papua New Guinea. Length variation in *pfhrp2* and *pfhrp3* was also found in the global *P. falciparum* population (Fig. 4). The lengths of the global *pfhrp2* and *pfhrp3* mainly ranged from 200–300 and 100–200 amino acids, respectively. The mean amino acid length of global *pfhrp2* was 245.2 ± 41.9, whereas the value for global *pfhrp3* was 178.2 ± 31.7. Diverse length polymorphisms between and among global isolates were found in both genes. The lengths of Myanmar *pfhrp2* varied from 50 to 350 amino acids (mean length 175.4 ± 70.3) and the size variation was greater than those of other countries analysed in this study. The mean lengths of *pfhrp2* from East Timor (253.7 ± 18.0), Solomon Islands (260.7 ± 23.4), Honduras (262.8 ± 14.3), Brazil (263.1 ± 12.4), French Guinea (286.8 ± 11.6), Nigeria (254.0 ± 17.3), Kenya (268.4 ± 19.7), Haiti (264.4 ± 14.4), Ghana (261.3 ± 11.0), Central African Republic (258.5 ± 14.4), Vietnam (271.0 ± 21.6), Thailand (255.6 ± 20.9), and Sri Lanka (260.3 ± 24.9) were longer than that of global *pfhrp2* (245.2 ± 41.9). The mean lengths of *pfhrp2* from Papua New Guinea (227.7 ± 40.0), India (240.3 ± 24.9), China-Myanmar border (212.4 ± 56.3), and Myanmar (232.3 ± 14.8) [25] were shorter than the mean length of global *pfhrp2*. The overall lengths of Myanmar *pfhrp3* were remarkably shorter than global *pfhrp3*. Most Myanmar *pfhrp3* were about 100 amino acids in length, with a mean length of 104.7 ± 30.4. The mean lengths of *pfhrp3* from Cambodia (268.6 ± 27.4), the Solomon Islands (168.4 ± 29.2) and Papua New Guinea (163.3 ± 21.8) were shorter than that of global *pfhrp3* (178.2 ± 31.7). In contrast, the mean lengths of *pfhrp3* from Colombia (211.8 ± 21.8), Peru (196.7 ± 14.5), Kenya (199.2 ± 17.6), and Philippines (184.7 ± 25.5) were longer than that of global *pfhrp3*.

**Table 2:** Frequencies of repeat types in global *pfhrp2*

Country	*n*	Type 1	Type 2	Type 3	Type 4	Type 5	Type 6	Type 7	Type 8	Type 9	Type 10	Type 12	Type 13	Type 14	Type 15	Type 19
Myanmar (This study)	84	96.4	100	69.4	36.4	41.2	92.9	78.8	64.7	-	88.2	95.3	50.5	10.6	-	-
Myanmar [25]	6	100	100	100	83.3	100	100	100	100	-	66.6	100	-	16.6	-	-
China	67	100	100	49.0	21.0	40.0	59.0	57.0	48.0	-	56.5	100	1.6	9.7	1.6	-
India	250	98.0	100	92.0	29.6	69.2	100	100	95.6	-	87.2	100	4.8	7.6	-	1.2
Sri Lanka	37	100	100	91.8	-	64.8	100	100	91.8	-	91.8	97.3	10.8	10.8	-	-
Thailand	7	100	100	75.0	33.3	41.6	100	100	91.6	-	83.3	100	33.3	-	-	-
Philippines	35	100	100	88.6	31.4	74.2	100	100	94.3	2.86	80.0	97.1	-	2.8	-	-
Cambodia	9	100	100	66.6	44.4	77.7	100	100	88.8	-	88.8	100	-	-	-	-
Vietnam	5	100	100	100	-	100	100	100	100	-	100	100	-	-	-	-
Central African Republic	13	92.3	100	92.3	23.1	84.6	100	100	92.3	-	92.3	100	15.4	15.4	-	-
Ghana	6	100	100	100	-	100	100	100	83.3	-	100	100	-	-	-	-
Haiti	7	100	100	71.4	57.1	57.1	100	100	100	-	85.7	100	28.6	-	-	-
Kenya	267	98.1	100	91.0	26.2	78.6	97.0	100	96.2	-	89.1	90.6	8.6	10.1	-	1.1
Madagascar	94	98.9	100	92.5	19.1	79.7	100	100	97.8	-	79.7	94.7	7.4	8.5	-	-
Nigeria	18	94.4	100	88.8	50.0	77.7	100	100	94.4	-	100	100	22.2	11.1	-	-
Tanzania	27	96.4	100	89.3	-	96.4	100	100	92.8	-	82.1	96.4	14.3	7.1	-	-
French Guinea	29	100	100	100	-	100	100	100	96.5	-	96.5	100	-	-	-	-
Brazil	8	100	100	100	12.5	87.5	100	100	-	-	25.0	100	-	-	-	-
Honduras	6	100	100	100	50.0	83.3	100	100	83.3	-	100	83.3	16.6	-	-	-
Papua New Guinea	31	93.5	100	93.5	19.3	83.8	93.5	96.7	90.3	-	74.2	96.7	12.9	9.6	-	-
Solomon Islands	19	100	100	100	-	73.6	100	100	100	-	94.7	94.7	5.2	10.5	5.2	-
East Timor	13	100	100	100	15.4	100	100	100	92.3	-	92.3	100	-	7.6	-	-

**Table 3:** Frequencies of repeat types in global *pfhrp3*

Country	*n*	Type 1	Type 2	Type 4	Type 7	Type 15	Type 16	Type 17	Type 18	Type 20	Type 28	Type 29
Myanmar (This study)	56	97.9	8.9	100	85.7	80.3	57.1	97.9	97.9	97.9	1.8	-
Cambodia	8	100	-	100	25.0	100	100	100	100	100	-	-
India	148	100	2.0	100	100	99.3	96.6	100	98.6	100	4.1	2.0
Philippines	7	100	28.6	100	100	100	100	100	100	100	-	-
Kenya	270	98.9	0.4	100	100	98.5	100	100	100	100	9.3	-
Madagascar	178	99.4	-	96.5	99.4	99.4	100	100	99.4	100	14.2	-
Nigeria	16	100	-	100	100	100	100	100	100	100	-	-
Peru	7	100	33.3	100	100	-	100	100	100	100	-	-
Colombia	5	100	-	100	100	100	100	100	100	100	20.0	-
Papua New Guinea	7	100	-	100	100	-	100	100	100	100	-	-
Solomon Islands	15	100	-	100	100	-	100	100	100	100	-	-

## Discussion

Malaria RDTs provide a simple, rapid and reasonably reliable diagnosis of malaria, which could aid proper treatment of the disease and offer significant benefits for malaria control and elimination. Given these advantages, global use of RDTs has been increasing rapidly in many malaria-endemic regions, and having RDTs with high specificity and sensitivity against global isolates is very important. However, genetic variation in the target antigens employed in the RDTs could affect the performance, especially their sensitivity to detect low-density malaria infections [9, 23, 25]. Understanding the genetic diversity and structure organization of the *pfhrp2* and *pfhrp3* in global *P. falciparum* isolates is important because most commercially available *P. falciparum* RDTs target PfHRP2 expressed solely by the parasite [26].

This study is the first report on genetic analysis of *pfhrp2* and *pfhrp3* in Myanmar *P. falciparum* isolates. Sequence analysis of Myanmar *pfhrp2* and *pfhrp3* suggested extensive genetic variations in the both genes consistent with previous studies for field isolates from various geographical areas [8, 9, 17, 22, 23, 25, 27, 28]. Similar to previous results, overall genetic diversity was greater in *pfhrp2* than *pfhrp3* in Myanmar isolates. Structural organizations of repeat types in Myanmar *pfhrp2* and *pfhrp3* were highly diverse. Most Myanmar *pfhrp2* and *pfhrp3* sequences occurred only once in the analysed *P. falciparum* isolates. Characteristics shared by the isolates were also identified. The majority of Myanmar *pfhrp2* started with type 1 repeat and terminated with type 12 repeat. Similarly, the majority of Myanmar *pfhrp3* started with type 1 repeat and terminated with type 4 repeat. This conserved structural organization was also identified in global *pfhrp2* and *pfhrp3* [25, 27–29]; the major repeat types found in Myanmar *pfhrp2* and *pfhrp3* were also the most common repeat types observed in global *pfhrp2* and *pfhrp3*. Although the frequency of each repeat type differed slightly in global isolates, repeat types 1, 2, 3, 6, 7, 8, 10, and 12 were the most common in Myanmar and global *pfhrp2* [25, 27–29]. The frequencies of repeat types 4, 5, 13, and 14 varied among global isolates. Repeat types 1, 4, 7, 15, 16, 17, 18, and 20 were the most common in Myanmar and global *pfhrp3* [25, 27–29]. Few repeat types were regional or country specific. For example, repeat types 15 and 19 were found only in limited numbers of *pfhrp2* from several countries including China, India, Kenya, or the Solomon Islands. Repeat type 29 was found only in India *pfhrp3*, and its frequency was low.

Although global *pfhrp2* and *pfhrp3* shared similar structural organizations, they also displayed differences. The most important differences identified were length polymorphisms, which are caused by variation in the number and arrangement of different repeat types. These length polymorphisms of global *pfhrp2* and *pfhrp3* may be induced by several molecular mechanisms, such as recombination, slipped-strand mispairing event, gene conversion, and unequal crossover [30–33]. The effects of length polymorphisms in *pfhrp2* and *pfhrp3* on diagnostic performance of PfHRP2-based RDTs are not clearly understood, but they could alter the binding affinity of specific monoclonal antibodies and consequently influence the sensitivity of PfHRP2-based RDTs [9, 23]. The relationship between the combined length of type 2× type 7 repeats in *pfhrp2* and the sensitivity of PfHRP2-based RDTs has been studied previously [8, 23, 25, 28, 34, 35]. Four studies [8, 23, 34, 35] proposed that at low parasitaemia (less than 250 parasites/µl), false-negative rates increased as combined lengths of type 2 × type 7 repeats decreased. In contrast, two studies [25, 28] found that sensitivity of PfHRP2-RDTs was not influenced greatly by combined lengths of type 2× type 7 repeats [25, 28]. Myanmar *pfhrp2* were classified into two major groups based on the lengths of type 2× type 7 repeats, borderline group (77.4%, less than 43) and group B (19%, ranged from 50 to 100). The impact of repeat length polymorphisms in Myanmar *pfhrp2* on the sensitivity of RDTs was not determined in this study because RDT results for all *P. falciparum* isolates were not available. Further studies to determine the effect of *pfhrp2* length polymorphisms on performance of PfHRP2-based RDTs is necessary. Amino acid changes in *pfhrp2* and *pfhrp3* are another important characteristic that cause genetic polymorphisms in global isolates [27, 36]. In total, 17 and 3 amino acid changes were found in Myanmar *pfhrp2* and *pfhrp3*, respectively, although the frequency of these changes was generally low. Most amino acid changes in Myanmar *pfhrp2* and *pfhrp3* were novel changes that have not been reported previously. The influence of amino acid changes identified in global *pfhrp2* and *pfhrp3* on the diagnostic performance of PfHRP2-based RDTs is also not clear yet, and therefore further study is required.

This study had several limitations. The *pfhrp2* and *pfhrp3* were not amplified successfully in all Myanmar *P. falciparum* isolates analysed in this study, a result explained by the poor quality of genomic DNA. Indeed, some of the *pfhrp2*-negative samples were also negative for *P. falciparum* merozoite surface protein-1 (*pfmsp-1*) and *pfmsp-2* amplifications. Otherwise, deletion of *pfhrp2* and *pfhrp3* in the negative samples is also a possibility, but further study to elucidate this is necessary. Although extreme sequence variations in Myanmar *pfhrp2* and *pfhrp3* were identified, the effect of these variations on the performance of PfHRP2-based RDTs was not elucidated clearly in this study. Further study, including larger sample sizes and RDT negative samples, is needed to understand the effect of genetic diversity and deletion of *pfhrp2* and *pfhrp3* on the performance of the PfHRP2-RDTs.

## Conclusion

Extensive genetic diversity was found in Myanmar *pfhrp2* and *pfhrp3*. Length polymorphisms due to variation in the number and arrangement of different repeat types as well as amino acid changes contributed to the genetic diversities of Myanmar *pfhrp2* and *pfhrp3*. Comparative sequence analysis of global *pfhrp2* and *pfhrp3* suggests that global *pfhrp2* and *pfhrp3* share similar structural features, but they also differ in some features. These results may provide a better understanding of the *pfhrp2* and *pfhrp3* structure in global *P. falciparum* population and suggest useful information to develop RDTs with improved quality. Further examination of genetic diversity of *pfhrp2* and *pfhrp3* in diverse global *P. falciparum* populations with a larger number of isolates also is necessary to better understand the structural nature of the two genes in the global populations.

## Supplementary information

**Additional file 1: Fig. S1.** Map of study site. Blood sample collection was conducted in four sites including Mandalay, Pyin Oo Lwin, Naung Cho, and Tha Beik Kyin in Upper Myanmar between 2013 and 2015.

**Additional file 2: Table S1.** Primers used to amplify *pfhrp2* and *pfhrp3.*

**Additional file 3: Table S2.** Accession numbers of *pfhrp2* sequences of global *Plasmodium falciparum* isolates enrolled in this study.

**Additional file 4: Table S3.** Accession numbers of *pfhrp3* sequences of global *Plasmodium falciparum* isolates enrolled in this study.

## Data Availability

The data supporting the conclusions of this article are provided within the article and its additional files. The original datasets analysed in this current study are available from the corresponding author upon request. The nucleotide sequences reported in this study have been deposited in the GenBank database under the accession numbers KX138275–KX138311, MG417056–MG417080, and MT591418–MT591439 for *pfhrp2*; KX138312–KX138340, and MT591440–MT591466 for *pfhrp3*.

## Abbreviations

PCR: Polymerase chain reaction
PfHRP2: *Plasmodium falciparum* Histidine rich protein 2
PfLDH: *Plasmodium falciparum* Lactate dehydrogenase
PfHRP3: *Plasmodium falciparum* Histidine rich protein 3
*pfmsp-1*: *Plasmodium falciparum* Merozoite surface protein-1
*pfmsp-2*: *Plasmodium falciparum* Merozoite surface protein-2
RDT: Rapid diagnostic test
rRNA: 18S ribosomal RNA
