# Supplementary figures and images for: Genetic variations in histidine-rich protein 2 and histidine-rich protein 3 of Myanmar Plasmodium falciparum isolates
Source: Malar J. 2020 Nov 2;19:388. doi: 10.1186/s12936-020-03456-6 (PMC7607715; doi:10.1186/s12936-020-03456-6)

## Slide 1
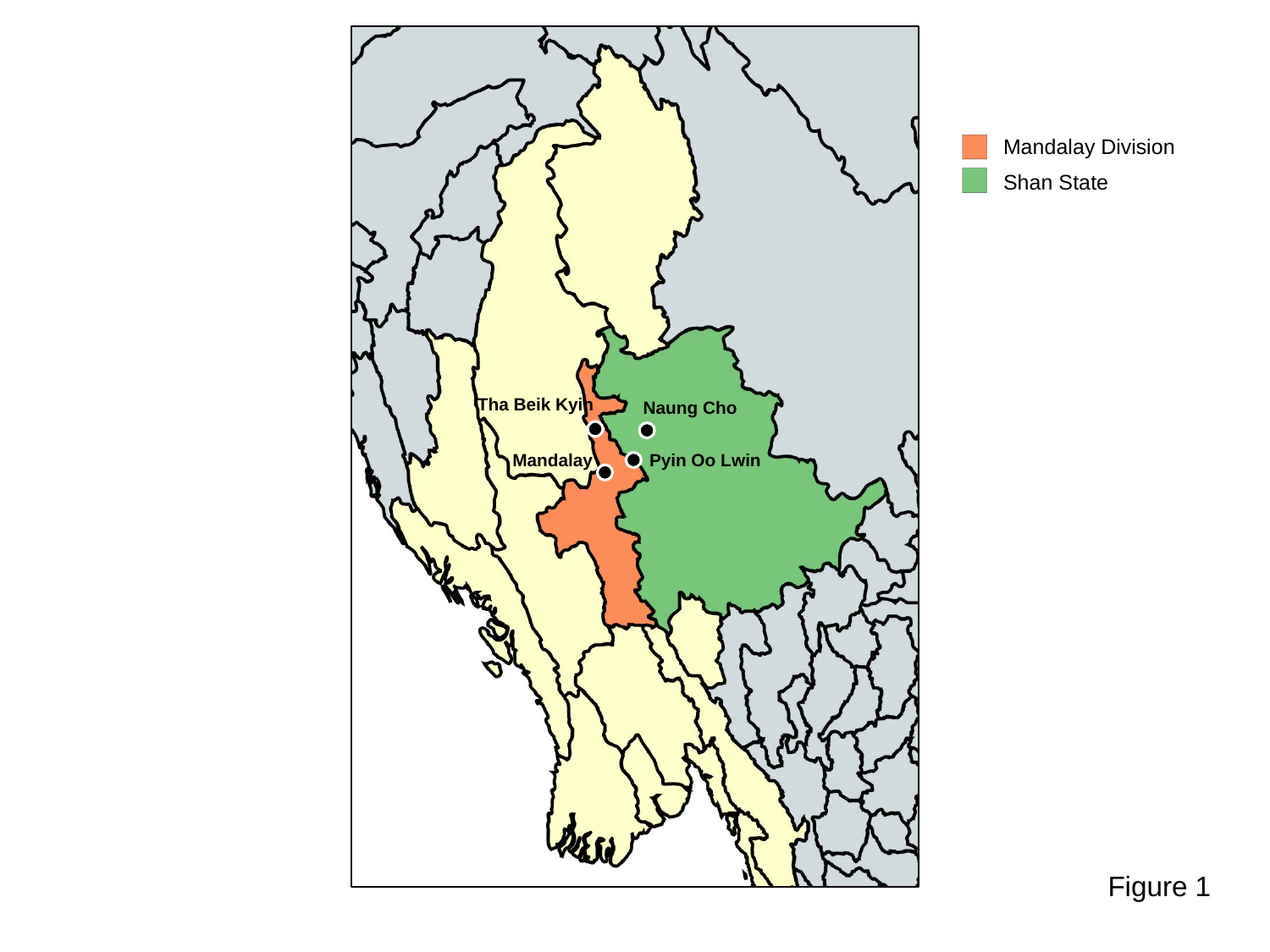

Mandalay Division
Shan State
Tha Beik Kyin
Naung Cho
Mandalay
Pyin Oo Lwin
Figure 1

Supplement: Supplementary file 1 — Additional file 1: Fig. S1. Map of study site. Blood sample collection was conducted in four sites including Mandalay, Pyin Oo Lwin, Naung Cho, and Tha Beik Kyin in Upper Myanmar between 2013 and 2015. [file 12936_2020_3456_MOESM1_ESM.pptx]
