# Supplementary material for: Genetic variations in histidine-rich protein 2 and histidine-rich protein 3 of Myanmar Plasmodium falciparum isolates
Source: Malar J. 2020 Nov 2;19:388. doi: 10.1186/s12936-020-03456-6 (PMC7607715; doi:10.1186/s12936-020-03456-6)
Supplement: Supplementary file 2 — Additional file 2: Table S1. Primers used to amplify pfhrp2 and pfhrp3. [file 12936_2020_3456_MOESM2_ESM.docx]

**Table S1 Primers to amplify *pfhrp2* and *pfhrp3***

| **Genes** | **Primer name** | **Primer sequence** |
| --- | --- | --- |
| *pfhrp2* | Pfhrp2-F | 5’-TGTGTAGCAAAAATGCAAAAGG-3’ |
|  | Pfhrp2-R | 5’-TTAATGGCGTAGGCAATGTG-3’ |
|  | Pfhrp2-NF | 5’-GCAAAAGGACTTAATTTAAATAAGAG-3’ |
|  | Pfhrp2-NR | 5’-GCAATGTGTGGCGGCTTCGTG |
| *pfhrp3* | Pfhrp3-F | 5’-AATAAGAGATTATTACACGAAAG-3’ |
|  | Pfhrp3-R | 5’-TGGTGTAAGTGATGCGTAGT-3’ |
|  | Pfhrp3-NF | 5’-ATTACACGAAAGTCAAGCACA-3’ |
|  | Pfhrp3-NR | 5’-GTGATGCGTAGTGGCAATATG-3’ |
