# Supplementary material for: Genetic variations in histidine-rich protein 2 and histidine-rich protein 3 of Myanmar Plasmodium falciparum isolates
Source: Malar J. 2020 Nov 2;19:388. doi: 10.1186/s12936-020-03456-6 (PMC7607715; doi:10.1186/s12936-020-03456-6)
Supplement: Supplementary file 3 — Additional file 3: Table S2. Accession numbers of pfhrp2 sequences of global Plasmodium falciparum isolates enrolled in this study. [file 12936_2020_3456_MOESM3_ESM.docx]

**Table S2. Global *pfhrp2* sequences analysed in this study**

| **Country** | **References** | **Accession numbers** |
| --- | --- | --- |
| Brazil (n=8) | [23,25] | AY816240, AY816292– AY816295, FJ871209– FJ871210, FJ871160 |
| Honduras (n=6) | [23,25] | AY816261, FJ871222– FJ871226 |
| Central Africa Republic (n=13) | [25] | FJ871174– FJ871186 |
| French Guinea (n=29) | - GenBank | KC558574– KC558602 |
| Ghana (n=6) | [23,25] | AY816251, AY816262, FJ871215– FJ871218 |
| Haiti (n=7) | [25] | FJ871221, FJ871227, FJ871345– FJ871349 |
| Tanzania (n=27) | [25] | FJ871373– FJ871399 |
| Kenya (n=267) | [25,27] | FJ871188– FJ871238, MH230283– MH230526 |
| Madagascar (n=94) | [25,35] | FJ871304– FJ871319, EU589688– EU589766 |
| Nigeria (n=18) | [23,25] | AY816309, FJ871327– FJ871340, FJ871371– FJ871372, FJ871362 |
| Papua New Guinea (n=31) | [23,25] | AY816241– AY816243, AY816254– AY816258, FJ871161– FJ871163, FJ871194, FJ871358, MF673786– MF673803 (GenBank) |
| Solomon Islands (n=19) | [23,25] | AY816268– AY816270, AY816296–AY816299, FJ871365– FJ871368, FJ871298– FJ871303, FJ871363– FJ871364 |
| Philippines (n=35) | [23,25] | AY816266– AY816267, AY816273–AY816291, FJ871212– FJ871214, FJ871341– FJ871344, FJ871354– FJ871357, FJ871359– FJ871361 |
| China–Myanmar border (n= 67) | [36] | KP712709– KP712775 |
| East Timor (n=13) | [25] | FJ871242– FJ871253, FJ871295 |
| Sri Lanka (n=37) | [25] | FJ871257– FJ871282 |
| Thailand (n=7) | [23] | AY816260, AY816263– AY816265, AY816300– AY816301, AY816308 |
| Vietnam (n=5) | [23] | AY816303– AY816307 |
| Myanmar (n=6) | [25] | FJ871321– FJ871326 |
| Cambodia (n=9) | [23,25] | AY816248, FJ871187, FJ871167– FJ871173 |
| India (n= 250) | GenBank | KX679582–KX679831 |
