# Supplementary material for: Genetic variations in histidine-rich protein 2 and histidine-rich protein 3 of Myanmar Plasmodium falciparum isolates
Source: Malar J. 2020 Nov 2;19:388. doi: 10.1186/s12936-020-03456-6 (PMC7607715; doi:10.1186/s12936-020-03456-6)
Supplement: Supplementary file 4 — Additional file 4: Table S3. Accession numbers of pfhrp3 sequences of global Plasmodium falciparum isolates enrolled in this study. [file 12936_2020_3456_MOESM4_ESM.docx]

**Table S3. Global *pfhrp3* sequences analysed in this study**

| **Country** | **References** | **Accession numbers** |
| --- | --- | --- |
| Cambodia (n=8) | [25] | GU194970- GU194976, GU194979 |
| Colombia (n= 5) | [25] | KC899083- KC899086 (GenBank), GU194987 |
| India (n= 148) | GenBank | KX679832- KX679968, KT238929-KT238939 |
| Kenya (n=270) | [25,27] | MH230527- MH230790, GU194977, GU194982- GU194986 |
| Madagascar (n=178) | [25,35] | EU589768- EU589942, GU194999, GU195003- GU195005 |
| Nigeria (n=16) | [25] | GU194993- GU194998, GU195015- GU195020 |
| Philippines (n=7) | [23,25] | GU195031, GU195024- GU195026, AY821806, AY821815, AY821824 |
| Peru (n= 6) | [25] | GU194966- GU194969, GU195027- GU195030 |
| Papua New Guinea (n=7) | [23,25] | GU195044, GU195011, AY821807, AY821813, AY821816, AY821823, AY821825 |
| Solomon Islands (n=15) | [23,25] | GU195034- GU195035, GU195006, GU195008, GU195010, AY821811, AY821817- AY821821 |
